# Development of Nanobodies Targeting Peste des Petits Ruminants Virus: The Prospect in Disease Diagnosis and Therapy

**DOI:** 10.3390/ani11082206

**Published:** 2021-07-26

**Authors:** Edson Kinimi, Serge Muyldermans, Cécile Vincke, Steven Odongo, Richard Kock, Satya Parida, Mana Mahapatra, Gerald Misinzo

**Affiliations:** 1 SACIDS Africa Centre of Excellence for Infectious Diseases, SACIDS Foundation for One Health, Sokoine University of Agriculture, P.O. Box 3297, Morogoro 25523, Tanzania; satyaparida1964@gmail.com; 2 Department of Veterinary Physiology, Biochemistry and Pharmacology, College of Veterinary Medicine and Biomedical Sciences, Sokoine University of Agriculture, P.O. Box 3017, Morogoro 25523, Tanzania; 3 Department of Veterinary Microbiology, Parasitology and Biotechnology, College of Veterinary Medicine and Biomedical Sciences, Sokoine University of Agriculture, P.O. Box 3019, Morogoro 25523, Tanzania; 4 Laboratory of Cellular and Molecular Immunology, Vrije Universiteit Brussel, Pleinlaan 2, 1050 Brussels, Belgium; Serge.Muyldermans@vub.be (S.M.); Cecile.Vincke@vub.be (C.V.); 5 Department of Biotechnical and Diagnostic Sciences, College of Veterinary Medicine, Animal Resources and Biosecurity (COVAB), Makerere University, Kampala 7062, Uganda; opodongo@yahoo.co.uk; 6 The Royal Veterinary College, University of London, Hawkshead Lane, North Mymms, Hatfield AL9 7TA, UK; Rkock@rvc.ac.uk; 7 The Pirbright Institute, Ash Road, Pirbright, Woking GU24 0NF, UK; mana.mahapatra@pirbright.ac.uk

**Keywords:** peste des petits ruminants virus, camelid, alpaca, nanobody, heavy-chain only antibody, diagnosis, therapeutics

## Abstract

**Simple Summary:**

Peste des petits ruminants virus (PPRV) causes a highly devastating disease, peste des petits ruminants (PPR) of sheep and goats, that threatens food security, small ruminant production, and the conservation of wild small ruminants. Current efforts are directed towards the global control and eradication of PPRV, an initiative of the World Organisation for Animal Health and Food and the Agriculture Organisation of the United Nations. A plethora of diagnostic tools for PPR were primarily developed for livestock. New innovative diagnostic tools are needed to detect PPRV in atypical hosts (e.g., *Camelidae*, *Suidae*, and *Bovinae*), in wildlife ecosystems, and in complex field situations. Recent studies confirmed that single-domain antigen binding fragments (nanobodies) derived from heavy-chain-only camelid antibodies have proven to be a powerful tool in diagnostics and therapeutics due to their unique properties, such as small size and strong antigen-binding affinity. Therefore, the main objective of this study was to generate PPRV-reactive nanobodies in order to set a pace for the development of diagnostic and possibly therapeutic nanobodies in the future. Initially, a strategy was developed whereby an alpaca was immunized with PPRV in order to raise an affinity-matured immune response, from which an immune nanobody library was constructed. Following phage display, nine nanobodies that specifically recognise PPRV were identified on enzyme-linked immunosorbent assay. This study has generated PPRV-reactive nanobodies and have significant implications in the development of cost-effective diagnostic tools in context with the planned eradication of PPR in the world.

**Abstract:**

Peste des petits ruminants virus (PPRV) causes a highly devastating disease, peste des petits ruminants (PPR) of sheep and goats, that threatens food security, small ruminant production, and the conservation of wild small ruminants in many developing countries, especially in Africa. Robust serological and molecular diagnostic tools are available to detect PPRV infection, but they were mainly developed for domestic sheep and goats. The presence of a wide host range for PPRV does present serological diagnostic challenges. New innovative diagnostic tools are needed to detect PPRV in atypical hosts (e.g., *Camelidae*, *Suidae*, and *Bovinae*), in wildlife ecosystems and in complex field situations. Interestingly, single-domain antigen binding fragments (nanobodies) derived from heavy-chain-only camelid antibodies have emerged as a new hope in the development of accurate, rapid, and cost-effective diagnostic tools in veterinary and biomedical fields that are suitable for low-income countries. The main objective of this study was to construct an immune nanobody library to retrieve PPRV-reactive nanobodies that enable the development of diagnostic and therapeutic nanobodies in the future. Here, a strategy was developed whereby an alpaca (*Vicugna pacos*) was immunized with a live attenuated vaccine strain (PPRV/N/75/1) to raise an affinity-matured immune response in the heavy-chain-only antibody classes. The nanobody gene repertoire was engineered in pMECS-GG phagemid, whereby a *ccdB* gene (encoding a lethal protein) was substituted by the nanobody gene. An immune nanobody library with approximately sixty-four million independent transformants was constructed, of which 100% contained an insert with the proper size of nanobody gene. Following phage display and biopanning, nine nanobodies that specifically recognise completely inactivated PPRV were identified on enzyme-linked immunosorbent assay. They showed superb potency in rapidly identifying PPRV, which is likely to open a new perspective in the diagnosis and possible treatment of PPR infection.

## 1. Introduction

Peste des petits ruminants virus (PPRV) causes a highly contagious viral disease, peste des petits ruminants (PPR) of sheep and goats, that poses serious socio-economic losses in the small ruminant industry [1,2]. The PPRV infection has been confirmed in both *Ruminantia* and *Camelidae,* as well as in *Suidae* [3,4,5,6,7]. Considering the importance of sheep, goats, and wildlife in the livelihood of more than 300 million farmers, landless villagers, and pastoralists in Africa, the Middle East and Asia, PPR causes food insecurity and poverty, and threatens biodiversity [4,8]. On an annual basis, PPR causes economic losses of the equivalent to around US $1.2 to 1.7 billion due to animal deaths, reduced production, and the cost of fighting the disease [9]. Approximately one-third of the financial losses occur in Africa and a quarter in South Asia [10,11]. However, current efforts are now being directed towards the PPR Global Control and Eradication Program (PPR GEP), an initiative of the global animal health community coordinated through the World Organisation for Animal Health (OIE) and the Food and Agriculture Organisation (FAO) of the United Nations [12,13]. It was estimated that an investment of US $7.1 billion on PPR GEP could be recovered within five years of successful eradication [11]. This gives an overall benefit–cost ratio of 33.8 for the most likely situation, which makes PPR eradication economically feasible [12,14]. However, some economists and scientists believe that the actual cost of eradication could be much lower than US $7.1 billion [11,12]. Unfortunately, the slowness of the response to PPR spread in disease-free zones and atypical host species (e.g., *Camelidae*, *Suidae*, and *Bovinae*) increases the likely eradication cost [12]. For instance, economic losses associated with the saiga antelope (*Saiga tatarica mongolica*) death toll in Mongolia, a critically endangered species in central Asia, was estimated at US $7.27 million [2].

Multiple wildlife and atypical host species can be infected with PPRV, which poses diagnostic challenges in multi-host system testing [3,15,16]. Commercially available serological and molecular diagnostic tools to detect PPRV infection were mainly developed for domestic sheep and goats [17,18,19]. Thus, accurate diagnosis and standard protocols for interpretation of PPR diagnostic tests in atypical host species need to be established. For instance, previous studies showed that the haemagglutinin protein (H)-based competitive enzyme-linked immunosorbent assay (H cELISA) has a lower sensitivity in cattle compared to domestic sheep and goats [20,21,22,23]. The differences between PPRV H cELISA and neutralisation tests in buffalo sera have also been reported, indicating that differential antiviral immune responses among host species may affect the serology and interpretation of results [24]. The serological tool spectrum for PPR diagnosis (Virus Neutralization Test, immunochromatographic lateral flow devices, blocking ELISA, pseudotype-based neutralization assays, and PPR-Luciferase Immunoprecipitation System) have inherent strengths and weaknesses that require parallel optimization and validation [17,25]. Diagnostic tools to detect active infection, such as antigen ELISA and reverse transcriptase polymerase chain reaction (RT-PCR), are critical to the prompt implementation of control measures [26]. The presence of in-depth genomic information has the potential to clarify the roles of wildlife and domestic animals in PPRV circulation, viral evolution, and direction of transmission at wildlife-livestock interfaces. This highlights the need to develop diagnostic tools and current protocols need to be standardized and adequately validated for atypical species affected and type of sample collected [15,27].

The access of required diagnostic tools, vaccines, and therapeutics are limited or available in short supply in distant centralized laboratories in low-income countries [18,28]. This is also compounded by the restrictions in transporting clinical samples across international borders for confirmation and further studies of PPRV in the OIE reference laboratories, due to the Convention on International Trade in Endangered Species of Wild Fauna and Flora, and Nagoya Protocol regulations [15,24]. Thus, deployment of cost-effective technology to break the limitations in the development of novel innovative diagnostic and therapeutic tools for PPR in the developing world can be of additional value to the Global PPR Control and Eradication Program [17]. Recent advances in technologies such as material sciences, genomics, biotechnology, nanotechnology, and microfluidics provide opportunities to develop cost-effective diagnostics and therapeutics reagents for PPR, suitable for resource-limited settings [17,29]. The development of vaccines, diagnostics, and therapeutics greatly rely on detailed insights into the PPRV genome [30,31]. Like any member of Morbillivirus, the genome of PPRV is organised into six genes in the order of 3′-*N*, *P*(*C*/*V*), *M*, *F*, *H*, and *L*-5′, and each of these genes code for a distinct protein [30,32]. The encoded proteins bear the acronym of the respective gene of origin: nucleoprotein (N), phosphoprotein (P), matrix protein (M), fusion protein (F), haemagglutinin protein (H), and large polymerase protein (L) [33,34]. The *P* gene also codes for two additional non-structural proteins designated by C and V [35]. The N protein is abundant in PPRV-infected cells because the *N* gene is located near the genomic promoter and is hence the most transcribed gene [36]. Given its abundance and antigenic stability, the N protein has been a preferred candidate in PPR diagnostic development and the most appropriate gene for the molecular characterization of closely related isolates [37]. Most of the neutralizing antibodies are directed against the surface glycoprotein H [38,39]. For this reason, the N and H proteins are appealing targets in diagnostics and vaccine development, respectively [38].

A vital step towards eradication of PPR will be the cessation of vaccination and a switch to active surveillance in domestic and wild animals to identify the pock of endemicity responsible for PPRV persistence [40]. In this phase, active surveillance and disease reporting require robust and rapid diagnostic tests, which provide pen-side diagnosis [19,41]. Moreover, the lessons learnt from rinderpest eradication in 2011 meant that rapid and simple diagnostic tests based on monoclonal antibodies were available in the last phase of rinderpest eradication [41,42]. These tests were developed based on innovative diagnostic technologies that include Clearview chromatographic strip tests for rinderpest (Unipath, Bedford) and improved chromatographic strip tests for rinderpest and PPR detection (Svanova Biotech) [42]. The latter test recognised a wider range of rinderpest virus strains, including several strains of lineage 2 which had proved difficult to detect previously by the Clearview device [42]. In the final phase of PPR eradication, the development and use of serology that can differentiate vaccinated from naturally infected animals (DIVA) may play a significant role in controlling PPR outbreaks, enabling detection of cryptic foci, inadequate vaccine deployment, and other challenges in the midst of an eradication campaign. Thus, continued research funding is necessary to improve existing diagnostic tests, vaccines, and use of new innovative technologies, such as nanobodies, the Oxford nanopore MinION sequencer and the DIVA vaccine, to handle complex epidemiological situations that may arise during eradication [17,29].

Nanobody technology has emerged as a new hope in the development of accurate, rapid, and cost-effective diagnostic tools in veterinary and biomedical fields that are suitable for low-income countries [43,44,45,46,47]. A nanobody is the single-domain antigen binding fragment (12–15 kDa) of heavy-chain-only antibodies derived from *Camelidae* blood, devoid of light chains [48,49]. The nanobodies have proven to be powerful tools in diagnostics and therapeutics due to their unique biophysical, biochemical, and pharmacological signature advantages [50,51]. In particular, the recombinant expression of nanobodies in microbial systems and straightforward purification using His-tag by immobilised metal affinity chromatography makes their purification easy and at an affordable cost [49,52]. Nanobodies are generated at a large scale in bacterial systems or lower eukaryotes with superiority, which is crucial for their use in diagnostics and therapeutics [51,53]. Moreover, nanobodies are thermally stable, soluble, and ten times smaller than classical antibodies, and can be easily generated using the golden gate cloning strategy [54]. The golden gate cloning strategy generates a high-quality immune nanobody library by employing pMECS-GG phagemid in cloning whereby a *ccdB* gene (encoding a lethal protein) is substituted by the nanobody gene [54]. In this strategy, a considerably large immune library is produced where 100% of the clones possess a phagemid carrying an insert with a length of a nanobody gene [54]. The immunization, bleeding for peripheral blood, lymphocyte preparation, and cDNA synthesis are all performed in the golden gate cloning strategy [52,54]. Thus, the immune library can be constructed within a week and is more cost-effective than previous standard approaches using classical restriction enzymes and ligations [52,55].

The present study was carried out to generate PPRV-reactive nanobodies, so that nanobodies with diagnostic and therapeutic applications could be developed in the future. The availability of PPRV-specific nanobodies provides an opportunity for the development of rapid and accurate diagnostic tests and with perspective for therapeutic purposes.

## 2. Materials and Methods

### 2.1. Antigens and Antibodies

The live attenuated PPRV/N/75/1 vaccine strain was outsourced from Botswana Vaccine Institute, Botswana for immunisation of the alpaca. Whole killed PPRV antigen mixture generated from completely inactivated PPRV was obtained from The Pirbright Institute, United Kingdom for affinity selection of PPRV-reactive nanobodies. For enzyme-linked immunosorbent assay (ELISA) tests, rabbit anti-camel VHH antibody, goat anti-rabbit-horseradish peroxide (HRP, BioRad, Hercules, CA, USA), anti-M13-HRP, mouse anti-His tag antibody, and goat anti-mouse alkaline phosphatases (Sigma-Aldrich, St. Louis, MO, USA) were all provided by Vrije Universiteit Brussel, Brussels, Belgium and all were used according to the manufacturer’s instructions.

### 2.2. Short Immunisation Scheme

An adult alpaca was subcutaneously injected in the shoulder with 1 mL of live attenuated PPRV/N/75/1 vaccine strain at ≥ ×10^2.5^ TCID_50_ per dose in two-week intervals (day 0, 14 and 28). Blood was collected from the jugular vein on day 40.

### 2.3. Nanobody Library Construction

On the 40th day from the start of the immunisation, 50 mL of blood was taken, and peripheral blood lymphocytes (PBLs) were purified on Leucosep^®^ tubes (Greiner Bio-One, Monroe, NC, USA). An immune nanobody library was constructed as previously described [54,56]. In brief, total mRNA isolated from PBLs was used as a template to synthesise cDNA using oligo dT primers. The cDNA was subsequently amplified with a variable-domain heavy chain leader-specific primer CALL001 and a CH2-specific primer CALL002 to amplify the heavy chain gene fragments from the variable region to the CH2 region from conventional and heavy chain-only antibodies, as previously described by Romão et al. [54]. The resulting first PCR amplicons with lengths of approximately 700 bp (which contain the *VHH* of heavy chain only camelid antibodies (HCAbs) and 1000 bp (which contain the *VH* of the convention IgG) were separated by agarose gel electrophoresis. The 700 bp fragment was cut out of the gel with a scalpel blade and purified using QIAquick gel extraction kit (Qiagen, Hilden, Germany). Second PCR was performed with VHH-BACK SAPI, which anneals at the *VHH* template strand and introduces the *Sap*I recognition sequence in VHH-genes family-3 whilst VHH-FORWARD SAPI hybridizes with the *VHH* coding strand and also has a *Sap*I recognition sequence, as previously described [54]. The resulting amplicons were purified, cut with the *Sap*I restriction enzyme and ligated in the phagemid vector pMECS-GG in frame with a hemagglutinin (HA)-tag and a His6-tag, and transformed in electro competent *Escherichia coli* TG1 cells, as previously described by Romão et al.

### 2.4. Biopanning and Screening of PPRV-Reactive Nanobodies

Biopanning is an affinity selection technique in which specific binders (i.e., peptides, antibodies, nanobodies) against a target of choice are enriched from a phage display library during consecutive cycles of incubation, stringent washing, amplification and re-selection of bound phages [57,58]. Several rounds of affinity selection (biopanning) and washing away unbound phages are necessary to enrich specifically binding phage particles [53,59]. In this study, four rounds of panning were performed to enrich PPRV-specific nanobodies, as previously described [53]. The nanobodies from the library were displayed on phage particles after M13K07 helper phage infection of the *E. coli* TG1 cells. An aliquot (1 mL) of the cloned library with a complete nanobody repertoire (at least 100× the library size) was grown to exponential phase before superinfection with M13 helper phage (20× excess of bacteria). Phage particles were then recovered through precipitation with sodium chloride polyethylene glycol solution and subjected to four rounds of panning on solid phase coated with whole killed PPRV antigen mixture (2 µg/well). The PPRV antigen-bound phage particles were eluted by adding 100 mM Triethylamine, pH 11.5, neutralised by 1.0 M Tris-HCl, pH7.4. The neutralised solution with eluted phage particles was used to infect *E. coli* TG1 cells. Parts of infected cells were used in subsequent rounds of selection on 2xTY/AMP-KAN (AMP is ampicillin and KAN is Kanamycin) and the rest were used for evaluation of enrichment on LB-AMP/GLU agar plates. Afterwards, independent colonies were grown on a master reference plate, cultured, and expressed. Expression of the nanobody protein was induced overnight in the presence of 1 mM isopropyl-ß-D-1-thiogalactopyranoside (IPTG) (Sigma-Aldrich, St. Louis, MO, USA). After extraction by osmotic shock, the periplasmic extracts containing the nanobodies were added to wells of a microtiter plate coated with PPRV antigens (1 µg/well). The presence of PPRV-specific nanobodies was detected in an ELISA using a mouse anti-His antibody and anti-mouse alkaline phosphatase (BioRad, Hercules, CA, USA). The VHH gene inserts in pMECS-GG of colonies scoring positive in ELISA were sequenced and analysed. The pMECS-GG vector containing unique PPRV-specific nanobodies were then transformed and expressed in the non-amber codon suppressor *Escherichia coli* WK6 cells. Soluble nanobodies from the periplasmic extract were tested in ELISA for their capacity to recognize native PPRV antigens.

### 2.5. Enzyme-Linked Immunosorbent Assay

Native PPRV antigens were coated in test wells (1 µg per well) in a 96-well plate (Maxisorp Nunc) and were incubated overnight at 4 °C in cold room. The plate was washed five times with 300 µL of phosphate buffered saline (PBS) containing 0.05% Tween-20 (PBS/Tween-20) in each step and blocked for 2 h at room temperature with 200 µL of 2% skimmed milk powder in PBS in both test and control wells. Periplasmic extract nanobodies of 100 µL were added into each test and control well and incubated for 1 h at an ambient temperature. Then, the plate was emptied and washed five times to remove unbound and excess periplasmic extract. Anti-PPRV soluble nanobodies were detected by adding 100 µL of primary antibody (mouse anti-His tag antibody diluted in 1/2000 blocking solution) in each well. The reaction was incubated for 1 h at an ambient temperature. The wells were emptied and washed five times to remove the unbound primary antibody, followed by adding 100 µL of conjugate secondary antibody (anti-mouse alkaline phosphatase antibody diluted in 1/2000 blocking solution) into each well and incubating at an ambient temperature for 1 h. The ELISA plates were developed by adding 100 µL of freshly prepared *p*-nitrophenyl phosphate disodium salt solution (0.06 g in 30 mL of distilled water). The results were read at a wavelength of 405 nm. Thus, a colony was considered “positive”, i.e., expressing a nanobody that recognizes PPRV antigens in ELISA, when an absorbance in the antigen coated well was at least twice that of the well without the antigen for the same periplasmic extract, as previously described by Vincke et al. [53].

## 3. Results

### 3.1. Nanobody Library Size

A nanobody gene pool containing the original diversity of the antigen-binding domains of the HCAbs was generated from 2.26 × 10^8^ lymphocytes. These nanobody genes were ligated in the pMECS-GG phage display vector. Thus, following electro-transformation in TG1 cells and selection on absence of cytotoxic ccdB protein and presence of ampicillin resistant colonies, we obtained a considerable large nanobody library of 6.4 × 10^7^ independent transformants.

### 3.2. Enrichment of Nanobody Library

The PPRV nanobody binders were generated on four consecutive rounds of in vitro selections in ELISA plates coated with completely inactivated PPRV whole antigen. Using this strategy, a clear PPRV-specific enrichment was observed from the second round of panning onwards with an approximately hundred-fold enrichment, as previously described [60].

### 3.3. Selection of PPRV Reactive Nanobodies

**Figure 1:**
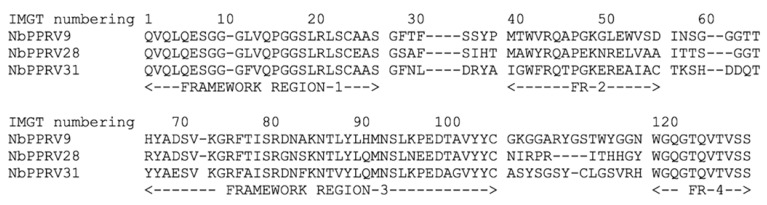
Alignment of single domain antibody sequences of three nanobody families that recognise peste des petits ruminants virus antigens in enzyme-linked immunosorbent assay based on complementarity determining regions (CDRs) of nanobody.

**Figure 2:**
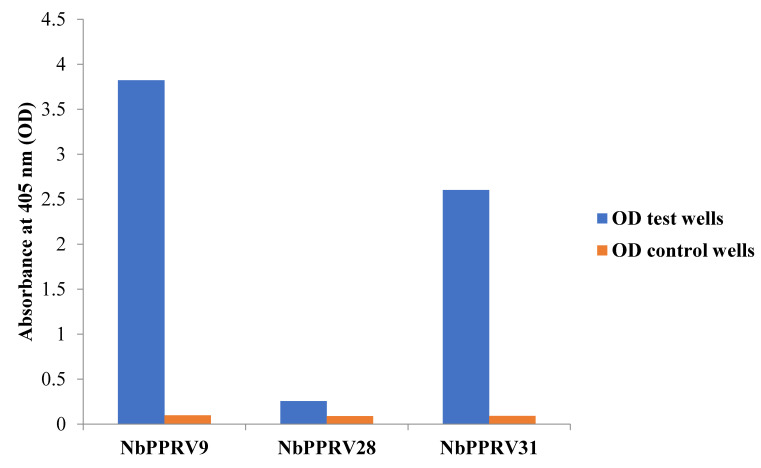
The bar chart represents an optical density of potent nanobodies directed against peste des petits ruminants virus (PPRV) in indirect enzyme-linked immunosorbent assay (ELISA). These nanobodies were rapidly detecting PPRV antigens with distinct signal strength in each nanobody family upon development of ELISA.

Four rounds of panning enriched the phage particles with anti-PPRV antigen-specific nanobodies. Ninety-four individual colonies from round two to four of panning were screened for the presence of nanobodies that recognise native PPRV antigens. Nine nanobody clones that were positive in ELISA for PPRV recognition were obtained. The PPRV-reactive clones were sequenced and the resulting VHH inserts were classified into families based on different complementarity determining regions (CDRs). The obtained clones belonged to three families based on their complementarity determining region-3 (CDR3) (Figure 1). The nanobody clones represented by NbPPRV9 had an imprint in their framework-2 region (the conserved region between CDR1 and CDR2) that resembles a VH of a classical antibody. It contains V42, L50, and W52 hallmark amino acids (numbering according to IMGT). The two other clones clearly have a VHH framework-2 imprint with Y42 or F42, R50, and L52 or A52. One of these nanobodies (clone NbPPRV31) contains an interloop disulphide bond between C55 and a C in the middle of its CDR3 (Figure 1). After expressing the nanobody proteins in non-amber suppressor WK6 cells with a C-terminal HA-tag and His6-tag, three families of PPRV-specific nanobodies periplasmic extract were further tested on indirect ELISA to detect PPRV antigens. They demonstrated clear rapid detection signals on ELISA plate upon development (Figure 2).

## 4. Discussion

Following the PPR Global Control and Eradication Strategy, an initiative of the OIE and the FAO of the United Nations, diagnosis remains the cornerstone towards the implementation of appropriate control measures including quarantine, vaccination, and possible stamping out [9]. Robust serological and molecular diagnostic tools are available to detect PPR infection, but they were mainly developed for domestic sheep and goats [17,18,19]. The presence of a wide host range does present diagnostic challenges [24]. Thus, deployment of cost-effective technology to break the limitations in the development of novel diagnostic and therapeutic tools for PPR is critical for effective surveillance of PPR in wildlife and atypical host species [17]. Interestingly, the rapid technological advances in areas such as material sciences, genomics, nanotechnologies, and microfluidics provide opportunities to develop cost-effective diagnostics and therapeutics reagents for PPR [17,29]. The applications of nanobodies in diagnostics and therapeutics are exponentially growing in biomedical and veterinary fields [46,61,62,63]. A very limited number of and extremely expensive biological antiviral treatments are available to control PPRV infections in sheep and goats [64,65,66]. The PPR antivirals cost high from the perspective of animal production [64,65,66,67]. For the same economic reasons, there is no OIE-prescribed veterinary antiviral curative treatment to fight against PPRV in infected animals [68,69]. The only treatments used are preventive vaccinations using live attenuated vaccines, including Nigeria/PPRV/75/1, Sungri 96, Arasur 87, and Coimbatore 97 [70,71]. The present study was carried out to generate nanobodies directed against PPRV, so that nanobody-based assays and therapy could be developed in the future.

In the present study, three nanobody families were identified from the immune nanobody library that are rapidly recognising PPRV antigens in ELISA (Figure 1). The nanobodies within the same family share similar CDR3 sequences, but might differ from each other by several point mutations, mainly spread between CDR1 and CDR2. The variation of the binding intensity between these nanobodies in ELISA may be due to different in expression of nanobodies in bacterial cells or varying affinities to the target PPRV antigens. Similarly, previous research has demonstrated that if the concentration of the nanobody is not normalised, the intensity of the ELISA signal is a function of both the expression level of the nanobody and the affinity of the nanobody–antigen interaction [55]. These potent nanobodies directed against PPRV antigens may mark the beginning of the use of nanobodies as analytical tools for the diagnosis and possible therapy of PPRV infection in the future. Thus, this study confirms the practicality of isolating a panel of PPRV-specific nanobodies from an immunised alpaca without having prior knowledge of the antigens involved, as previously reported [72]. The rapid detection of PPRV antigens with nanobodies that exert strong binding signals in ELISA is likely to open new perspectives in the diagnosis or therapy of PPR, as demonstrated in recent studies, including the coronavirus disease 2019 (COVID-19) [43,46,50,73]. Thus, further characterization of PPRV-reactive nanobodies is required to determine their binding affinities, target PPRV proteins, and neutralisation potential. For diagnostic purposes, the reactivity against the N protein will be sufficient, but for a therapeutic application, the virus neutralisation based on the surface glycoproteins H or F reactivity must be available [19,74,75].

Previous studies demonstrated that PPRV-infected camels raise strong immune responses and develop an active clinical syndrome [6,7,76]. It was clear that an immunised alpaca provides direct access to the in vivo affinity-matured antibodies, an advantage to identify highly specific and affinity-matured nanobodies. Thus, the blood of this immunised alpaca can be a good source to clone the nanobodies for subsequent selection of a panel of PPRV-specific nanobodies with diagnostic and therapeutic potential. Following the recent advances in nanobody production through the golden gate cloning strategy, an immune library can be cost-effectively constructed within a week [54]. In fact, panning on PPRV antigens yielded a high and clear enrichment of phage nanobodies from the second round of panning onwards, as previously reported [60]. This is considered as a clear indication for the abundance of PPRV antigen-specific binders in our immune library. Furthermore, it has been repeatedly demonstrated that the phage nanobodies are readily amenable to produce soluble and highly expressed monomeric binders [52,60]. The PPRV nanobody library constitutes a source of nanobodies directed against PPRV antigens.

The diagnosis of PPR based on ELISA is available to assess seropositivity within sheep and goats, with high sensitivity that detects antibodies to either the N or the H proteins of the virus [77]. However, the presence of a wide host range does present diagnostic challenges; current protocols need to be validated for atypical species affected, and the need to improve diagnostic tests is highlighted [25]. New diagnostic tools are needed to detect PPR infection in atypical hosts in wildlife ecosystems and in complex field situations [26]. The availability of PPRV-specific nanobodies provides an opportunity for the development of rapid and accurate diagnostic tests and possibly therapeutic nanobodies. We envisage that these potent nanobodies with the capability for binding native PPRV antigens may play a significant role in controlling PPR outbreaks by enabling detection of cryptic foci and addressing inadequate vaccine deployment and other challenges in the midst of PPR GEP operations. Further studies are necessary to decipher the possible optimum detection combination of these nanobodies and their structural functional relationship for progression to developing a nanobody-based pen-side test for PPRV. A proof-of-concept experimental investigation yielded proficient nanobodies against native PPRV antigens that could enable the development of diagnostic and therapeutic nanobodies in the future. The use of novel innovative technologies such as PPRV-reactive nanobodies can be an additional diagnostic and therapeutic tool in context with the planned eradication of PPRV in the world.

In most cases, vaccines, prophylactics, therapeutics, and reliable diagnostic tools are largely inaccessible, absent, or available in short supply in distant centralized laboratories in developing countries [18,28]. Compared with other biologics, classical monoclonal antibodies produced with long-stablished hybridoma technology are acceptable as the gold standard in immunotherapy and diagnostics [78,79,80]. However, these classical monoclonal antibodies need more support costs and they are difficult for massive production compared to their counterpart nanobodies [81,82,83]. The high production cost of classical monoclonal antibodies, limited tissue penetration, and less favourable pharmacokinetic stability have stimulated the use of smaller alternative antibody formats, such as the antigen binding fragments, single-chain variable fragments, and nanobodies [84,85]. Nanobodies have proven to be powerful tools in diagnostics and therapeutics due to their unique properties such as small size, strong antigen-binding affinity, high stability, water solubility, and preferential binding to cavities or grooves on the surface of the antigen, and resistance to extreme conditions (pH, pressure, chaotropic agents or proteases), often assisted by an extra interloop disulphide bond [62,86]. Furthermore, nanobody proteins are robust against thermal denaturation, which obviates a cold chain for transport and storage, suitable for the hot climate in sub-Saharan Africa, where cold-chain is unreliable. The low cost of high quality and the robustness of nanobodies will be an important feature for the development of cheap and sensitive diagnostic kits, either as lateral flow devices or as electrochemical detection assays suitable for low-income countries [44,45].

## 5. Conclusions

In conclusion, this study confirmed that PPRV-reactive nanobodies can be retrieved from an alpaca immune nanobody library. These proficient nanobodies against PPRV could open a new possibility in the diagnosis, vaccination, and treatment of PPR infection. Further studies need to be conducted to optimise the PPRV-potent nanobodies and determine their specificity and sensitivity to PPRV field isolates in comparison to other OIE prescribed diagnostic tests.

## Data Availability

The genetic data sets of PPRV-reactive nanobodies were presented in this article. The following Springer Protocols used in this study; Construction of High-Quality Camel Immune Antibody Libraries by Ema Romão et al., 2018 and Generation of Single Domain Antibody Fragments Derived from Camelids and Generation of Manifold Constructs by Cécile Vincke et al., 2012.
